# Nitrous Oxid Gas

**Published:** 1902-11-15

**Authors:** S. Straith


DENTAL REGISTER.

Vol. LVI. November 15, 1902. No. 11.

### NITROUS OXID GAS.

BY S. STRAITH, D.D.S.

Read before the Southwestern Michigan Dental Society, September 10, 1902.

In attempting to sustain the long-standing and well-deserving high reputation of nitrous oxid as the ideal anesthetic for the dentist, I do not wish to do so by any effort to tear down any *deserving* reputation or praise due to any other anesthetic, local or general. On the contrary I ever wish to be found ready to listen to, and profit by, favorable results obtained from the use of any other. Experimentation never was a hobby of mine and I have consequently been content with using the first born of all, nitrous oxid. However, I must plead guilty to using cocain in my first two years of practice, later to a short trial of chlorotone and then to eucain, which anesthetic I keep in my cabinet and occasionally (very rarely) use; and for extractions only when my patient can not be prevailed upon to use gas.

On the eleventh of December, 1894, at Philadelphia, occured the memorial exercises of the fiftieth anniversary of the discovery of the greatest boon to humanity that has ever existed—that of anesthesia—by Dr. Horace Wells; and the anesthetic *nitrous oxid.*

While Priestly, in 1776 discovered this gas, and Davy in 1800 noticed its exhilarating and analgesic effects, yet it was the province of Dr. Wells, a dentist, to prove and to announce to the world its true anesthetic value in 1844, by himself submitting to the experiment of having a tooth extracted while under its influence. Ever since that date, nitrous oxid, as well as every other good thing does, has undergone experiment after experiment to improve upon it, supplant or supersede it by something better, but, nitrous oxid is as pure and simple to-day, as it ever was, stands upon the highest round of the ladder containing the records of the comparative safety and efficiency of all anesthetics ever used.

Nitrous oxid has lived to see many an anesthetic plant sprout, grow and thrive for a time under the warmth of advertising or recommendations by its "discoverer" and his deluded followers, and then wither, die and sink into oblivion, leaving very few mourners (except the patients who submitted to its use).

When a patient presents himself for the extraction of a tooth, and the use of an anesthetic is desired or indicated, our first and last thought should be for the safety of the patient. Accordingly we ought to never let the matter of convenience influence us in the selection of an anesthetic, and our choice should not fall upon one whose composition is a secret; for, not knowing its constituent parts, if alarming symptoms arise from its use, we are at a loss to know what has caused them and consequently are unable to apply remedies for their relief. Of course, we are always told that "my preparation has always given success and never an alarming symptom,'' but I insist that we are not justified in using any anesthetic whose ingredients, and their possible if not probable effects upon the system are not known and generally understood and remedies for their relief at hand. If we disregard this, we are placing the life of our patient in jeopardy without sufficient grounds for our defense.

If safety, then, is to be the first consideration in our selection of an anesthetic, to what must we turn for evidence of that safety? Can we find a better source than the record of thousands upon thousands of administrations without the loss of life and the *exceedingly* small percentage of ''alarming symptoms'' or ''after effects?'' In our search for these statistics we always have found and do find nitrous oxid at the top of the list. Dr. Nevins, an extracting specialist in Chicago, with whom I am personally acquainted, says: ''I have administered gas to over 65,000 patients during the past thirty years. During the last ten years I have not refused to administer the gas to any patient no matter what his condition might be. I ask no questions and refuse no one; and never had an accident. If the gas is pure and properly administered, I believe it may be administered to patients of all ages, and I speak from experience; for I have given it to children under two years of age and to adults up to the age of ninety-two. I have gone to the homes of patients too sick to visit the office, and administered gas to them while in bed.'' Dr. Slonaker, another extracting specialist in Chicago, says: ''I have administered nitrous oxid gas to over one hundred thousand persons without an accident or unpleasant result. My opinion is that it is the safest anesthetic we can use.'' The Colton Dental Association of New York writes me as follows:

"NEW YORK, June 27, 1902.

"*Dear Doctor Straith:*—In reply to your letter I would say that we have given gas over two hundred and fifty thousand times in this office. I myself have given it fifty thousand times without a mishap. I give gas pure and without chloroform, ether or oxygen. I have used the A. C. E. mixture some with it, but prefer the nitrous oxid alone. I am always sure with pure nitrous oxid. All the gas given at Cooper Union for the past eight years has been given by Dr. McNeille.

"Fraternally yours,

"C. L. MCNEILLE."

These men all use nitrous oxid gas, pure and unadulterated, notwithstanding that Dr. Zigler says in the March number of the *Dental Summary* that "*pure* nitrous oxid gas is to-day never given by men who are looked upon as authority on the subject." As in the mercantile world, every substance that has proved its efficiency is constantly in competition with some adulteration or substitution, which is said to be "just as good" or "a little better," so in anesthetics. Nitrous oxid has been subjected to experimentation with ether, chloroform, alcohol, atmospheric air at different pressures and oxygen, and yet there are very few to-day who give nitrous oxid at all, who do not give it pure and simple.

Nitrous oxid is given because of its almost absolute safety, and when you mix with it any more dangerous substance you rob it of its safety and introduce danger just in proportion to the amount and quality of the adulterant. If you are not posted, ask your physician how much chloroform would be a dangerous dose for an exceptional person? Are you able to judge your patient's ability or inability to stand this small quantity of that powerful anesthetic? This small amount of chloroform if given *alone* might have no ill effects upon your patient, but if a smaller quantity be given after complete anesthesia is produced with gas, serious results might follow.

What leads you to introduce this more dangerous substance? I think I am safe in answering this question for you; you desire a longer anesthesia. Are you ready to answer for the consequences should you be so unfortunate as to administer this small quantity of chloroform to that person so susceptable to its powerful influence? Of course you have administered the mixture "hundreds of times" without the least trouble (to yourself) except the very occasional feeling of exhaustion and nausea or actual vomiting as an after-effect upon your patient. If you will notice the records of the few deaths that have occurred during, or following closely the administration of nitrous oxid, you will find that in most cases aside from the lodgement of some foreign body in the trachea, the gas used was mixed with some other substance. Refer to February, 1900, *Items of Interest*, for one instance.

Dr. Hewett has resurrected Paul Bert's theory of mixtures of certain amounts of oxygen with nitrous oxid, and advertises an apparatus, which he has "perfected," by the use of which he claims to get better results than with pure nitrous oxid alone. Dr. Hewett has a few followers, but unfortunately for him, ninety-nine percent of the dentists who use nitrous oxid at all, use it without the admixture of oxygen, so there has not been a ready sale for his perfected apparatus. He, and his followers, claim for their mixture that it does away with all of the "disagreeable symptoms" noticed when nitrous oxid is given alone, besides giving an average of fourteen seconds longer anesthesia. Some of the "disagreeable symptoms" mentioned are "cyanosia," "horrible screams" and cries, "muscular disturbances," etc., and as after-effects, headache, dizziness and nausea. Cyanosis is not a necessary adjunct to nitrous oxid anesthesia, as it succeeds and not *precedes* true anesthesia. The "horrible screams and cries," when they come, proceed from very nervous, excitable or hysterical people, but these outbreaks in my practice, have been so rare that I certainly would be unfair to put them down as an objection to the administration of gas. I do not recall an instance where these "screams" occurred during the *administration* of gas, but when heard were invariably due to an effort at extracting during the period of returning consciousness and they were never of so violent a character as to cause me to fear that a third eruption of Mount Pelee was about to overtake me.

Of the after-effects complained of, my observations have been that they are very conspicuous by their absence. Dr. Buxton in his work on anesthetics, says, that when nausea or vomiting follows the administration of nitrous oxid, it is due to the swallowing of gas, which distending, would cause a very sensitive stomach to react. Where a number of teeth have been extracted, or where hemorrhage is extensive, the swallowing of blood in greater or less quantities, is liable to occur and cause the stomach to rebel.

Dr. R. H. Cullum, of St. Paul, in his article on nitrous oxid and oxygen in the *Items of Interest*, May, 1900, claims as one of the advantages of this mixture the lengthened anesthesia in which he has been able to extract from five or six teeth, in one instance sixteen—at one administration, and in another instance he was able to extract all four third molars, with time to spare.

The writer, while making no claims at being a rapid extractor, has to his credit the accomplishment of the last-named fete, at least once, and in another instance, the removal of twenty teeth at one administration of nitrous oxid, pure and unadulterated. However, any one who is at all conversant with the use of gas knows that in the length of anesthesia produced, the conditions and circumstances must be *very favorable* if more than four or five to six or eight teeth are removed with one administration.

Dr. Ziegler says it demands a higher degree of skill and efficiency to use nitrous oxid and oxygen and. Dr. Hewitt's apparatus, than to use nitrous oxid alone, and later admits the patient is more liable to nausea and kindred after-effects, than when using pure nitrous oxid. The vast majority of dentists to-day are very seldom called upon to extract eight to ten or more teeth. Usually when such a case presents, the teeth are in a condition that a dentist who makes any pretensions at rapidity in extracting, are quite certain to succeed in removing all of them with one administration of pure gas. Yet in a few instances, I have been compelled to give it two or three times to remove that many teeth. I do not believe that ordinarily, more than that number of teeth ought to be extracted at one sitting.

Nitrous oxid, when given, should be absolutely pure, and my experience with gas manufactured by myself, leads me to believe that all the liquidfied gas supplied to us is not pure. While I used the liquidfied gas, I very seldom could produce complete anesthesia with less than from six and a half to eight or nine gallons, quite often using ten to twelve gallons and in a few instances fifteen or more gallons. With the gas of my own manufacture I use from four and a half to six gallons and very seldom as high as seven to eight gallons. Barring the admission of air through imperfect adaptation of face-piece, or imperfection in apparatus, which would be no more likely to occur in one instance than the other. I can not account for the excess of liquidfied gas used, in no way except it be from impurities. In the manufacture of nitrous oxid, with the description of which I will not tire you, but would say that I have here a drawing of my gas plant, and will be very glad to explain it to any one who may feel enough interested to ask questions after this session is over. If a heat of more than 500 degrees is applied, nitric oxid is formed, and if allowed to pass into the gasometer, adulterates the gas and also lessens its anesthetic powers, to say nothing of its injurious effects; and its presence may, and no doubt does account for some of the after-effects when they are noticed. Should this undesirable degree of heat be reached, it may produce five to ten or more gallons of this impure gas before the proper heat is restored and if allowed to pass into the gasometer, we have an impure gas, the use of which, in some instances, produces an unsatisfactory anesthesia. My gas plant is so arranged that if nitric oxid, which is noticed by white fumes, is being formed, I can, by simply closing a valve shut off its entrance into the gasometer and allow it to pass out into the room until the proper heat is restored and then again permit the gas to enter the gasometer. I do not believe gas becomes stale from age if properly stored; and cylinder gas five years old will give just as good results as if it had been used on the day of its manufacture. I would not have you understand that gas made by me is purer than that which could be made by any one else, but I do claim it to be purer than some I

have used. Pure gas, properly administered, will in thirty-five to fifty-five seconds produce anesthesia lasting from twenty-five to sixty seconds, depending upon the patient and this time is all that is necessary in the vast majority of cases to accomplish all that is indicated.

There are men who claim better results from the use of air with nitrous oxid, but my experience with it has been very unsatisfactory. I have never *tried* to administer air with gas, but on the contrary, the exclusion of air has been my aim. Sometimes in giving gas to men with a full beard, or from some other cause, there is an imperfect adaptation of the face-piece, and air is admitted, anesthesia may be produced in time, but is more liable to be followed by dizziness and nausea than when undiluted nitrous oxid is used. This mixture is apt to produce a state of anesthesia in which there is insensibility to pain, but not complete unconsciousness. I may cite one instance: A man about fifty-five years of age, wearing a full beard, presented for the extraction of the root of a superior lateral incisor. I administered about twenty gallons, and I do not know how much air through his beard, when I noticed that the conjunctival reflex was gone, and some evidence of cyanosis present. I ceased the administration and extracted the root, making three attempts before its complete removal was effected. The patient was quite slow in regaining consciousness and I was congratulating myself upon a very successful administration, when the patient awoke. I noticed that he appeared displeased and in answer to my question, "Well I guess you did not know much about the extraction of that tooth, did you?" said, "I knew it all! you could not hire me to take that stuff again. I could not move a muscle but knew everything you did. You might have extracted all my teeth and I could not have resisted you." Then he related to me what occurred, telling me just how many efforts I made to extract the tooth. I could relate similar instances.

There is on the market an inhaler with chloroform attachment, known as the Hurd Inhaler, having a small hood that fits over the nose, leaving the mouth free to admit air. I witnessed a very successful clinic with this inhaler, in Chicago, on the twenty-second of February, last; but noticed in a number of the cases that after the patient had breathed almost long enough to become indifferent to the anesthetic, a folded napkin was placed over the mouth, thus excluding the air, and he soon succumbed. This proved to me that air is not a *necessary* adjunct to nitrous oxid anesthesia, and that the inhaler, if used on a mouth breather, would be quite sure to asphyxiate the patient. I will be better able to judge of nitrous oxid and air anesthesia, with the Hurd Inhaler, when I see a demonstration of it without the chloroform attachment.

I would like to extend this paper further, for the more I say the more I want to say. Let one statement which can not be refuted, suffice: Nitrous oxid gas pure and unadulterated, and properly administered, has stood the test for nearly sixty years, and has to its credit the relief of more pain with less immediate or after bad effects than any other known anesthetic, local or general.

### DISCUSSION.

Dr. Hansen: If we wish to get the best results, the gas should be used free from air. This however, is easier said than done sometimes; for example, in "hatchet faces" it is a difficult matter to fit the face-piece closely. I used local anesthetics for about eight or ten years and went from this to the use of gas. The demands for it are not very frequent.

DR. RIX: I have had some experience in the use of nitrous oxid gas and I want to state right here I don't want anything to do with it. A man can not live longer on this gas than with any other subtle gas, and so far as excluding air is concerned, I say give them all the air you can. I think it is a very disagreeable anesthetic to administer. Of all the noises, screams, etc., why I have had them jump clear out of the chair. I have used chloroform and ether with good results a good many times, but not 50,000 or 100,000 times as the man mentioned in the paper. I doubt if any man ever administered gas to so great a number of people.

It is very necessary to teach the patient how to take the gas. Many times they are nervous and excited before coming into the office and are afraid to inhale the gas. The first thing is to get their confidence, after which it is an easy matter to instruct them how to inhale it.

I have produced anesthesia by the simple inhalation of air alone, enough so as to extract four teeth without a particle of pain.

There are several different apparatuses for the administration of gas. I used to manufacture my own gas pure and unadulterated, then I bought it from the manufacturers.

DR. WHITE: There is one thing I think in which Dr. Rix has made a mistake, and it is the administration of the gas in the presence of two people only. I think the proper thing is to always have the third person present.

In reference to the administration of gas, I don't believe that we as dentists have sufficient experience in the handling of the gas. We don't get enough gas extractions to warrant our keeping gas always on hand.

I think it would be advisable to have one man in each town to do all the extraction, of course this is not possible in small towns but it could be done in our larger towns and cities. I don't think any man should ever administer the gas who is afraid of it. If the patient notices any nervousness on the part of the operator he could not be expected to sit calmly in the chair and place himself under his care without some misgivings. In reference to the figures as stated in the paper about the number of administrations by a specialist of this kind, I don't think they are overdrawn, they keep an accurate account of all their work and these figures were placed on record and certified.

In reference to the gas to be used, I believe the best is that which is made by a reputable manufacturing establishment and placed in cylinders. I don't think any dentist should attempt to manufacture his own gas, it can not help but be impure. I endeavored to make my own for a while but after several explosions I gave it up.

Dr. Honey: The Hurd apparatus was referred to in the paper and from the manner in which it was presented a wrong impression might be obtained. The face-piece is made so as to fit over the nose, and is intended to be used while operating in the mouth, it is not possible to use it and carry out the idea of the inventor, by placing it over both mouth and nose. The patient can inhale the gas almost indefinitely, and while they are partly conscious, they are still insusceptible to pain. You can excavate teeth, cut down teeth for crowns and any other painful operation, without causing pain.

Dr. Hooper: Many times during the administration of gas the patients get very nervous before operation. I think if we could all understand the manner in which the gas is administered by these specialists, we would not doubt their skill and the number of administrations.

There are very few of the patients, one-half perhaps, who go to the office to have teeth extracted, who really know what they are going to take. They are sent there by other dentists to have the teeth extracted without pain and they do not question the method.

The patients who come into my office knowing I administer gas, are always in a nervous state, and it takes a great deal more gas and also time to handle such patients.

Another important point is to see that our apparatus is in good condition, having no leaky hose or broken face piece. This simple care may eradicate many difficulties with the gas and patient.

